# Not Early Referral but Planned Dialysis Improves Quality of Life and Depression in Newly Diagnosed End Stage Renal Disease Patients: A Prospective Cohort Study in Korea

**DOI:** 10.1371/journal.pone.0117582

**Published:** 2015-02-23

**Authors:** Ji In Park, Myounghee Kim, Ho Kim, Jung Nam An, Jeonghwan Lee, Seung Hee Yang, Jang-Hee Cho, Yong-Lim Kim, Ki-Soo Park, Yun Kyu Oh, Chun Soo Lim, Dong Ki Kim, Yon Su Kim, Jung Pyo Lee

**Affiliations:** 1 Department of Internal Medicine, Seoul National University College of Medicine, Seoul, Korea; 2 Clinical Research Center of End Stage Renal Disease in Korea, Daegu, Korea; 3 Department of Dental Hygiene, College of Health Science, Eulji University, Seongnam, Korea; 4 Department of Epidemiology and Biostatistics, School of Public Health, Seoul National University, Seoul, Korea; 5 Department of Internal Medicine, Seoul National University Boramae Medical Center, Seoul, Korea; 6 Department of Internal Medicine, Hallym University Hangang Sacred Heart Hospital, Seoul, Korea; 7 Kidney Research Institute, Seoul National University College of Medicine, Seoul, Korea; 8 Department of Internal Medicine, Kyungpook National University Hospital, Daegu, Korea; 9 Department of Preventive Medicine, Institute of Health Sciences, Gyeongsang National University of Medicine, Jinju, Korea; 10 N-Bio, Seoul National University, Seoul, Korea; Kaohsiung Medical University HospitalKaohsiung Medical University HospitalKaohsiung Medical University Hospital, TAIWAN

## Abstract

**Background:**

Health-related quality of life (HRQOL) has recently become an important issue. It reportedly affects morbidity and mortality in patients with end-stage renal disease (ESRD). In this study, we investigated whether early referral and planned dialysis improve the HRQOL and depression of patients with ESRD.

**Methods:**

We prospectively enrolled newly diagnosed patients with ESRD, from 31 hospitals in Korea, who completed questionnaires at 3 months after dialysis. We also got follow-up survey at 1 year after dialysis. To measure HRQOL and depression, Kidney Disease Quality of Life Short Form 36 (KDQOL-36) and Beck’s Depression Inventory (BDI) were utilized.

**Results:**

A total of 643 patients were analyzed. Referral type did not affect either KDQOL-36 or BDI scores. However, the planned dialysis group showed significantly better scores in 4 of 5 KDQOL-36 domains than did the unplanned group at 3 months after dialysis and partly, the effect was sustained for 1 year after dialysis. The benefit of planned dialysis was significant after adjusting for age, sex, type of dialysis, marital status, educational attainment, occupation, modified Charlson comorbidity index, albumin, and hemoglobin levels. BDI scores were also lower which indicate less depressive mood in planned dialysis group than those in unplanned group both at 3 months and 1 year after dialysis.

**Conclusions:**

Not early referral but planned dialysis improved both the short- and long-term HRQOL and depression of patients with ESRD. Nephrologists should try to help patients to initiate dialysis in a planned manner.

## Introduction

Health-related quality of life (HRQOL) of patients with end-stage renal disease (ESRD) is usually poorer than that of the general population. In addition, depression is a very common psychological problem in patients with ESRD [1]. These psychological issues should be considered as important as the physical problems because HRQOL and depression are reportedly related to morbidity and mortality rates in patients with ESRD [2–5].

In the last decade, timely nephrology referral in the predialytic stage of chronic kidney disease (CKD) has received great attention. Previous studies have revealed that the timing of referral makes significant differences in the mortality rate, hospital stay, type of dialysis, and medical costs [6–8]. The benefits of early referral might be attributable to the identification of reversible causes, specialized management, and optimal preparation of patient education regarding dialysis modalities [7]. Above all, preparation of dialysis access and optimal initiation of dialysis seem to be particularly important. One study revealed that the benefits of early referral are lost if dialysis is initiated in an unplanned manner [9].

The effect of early referral and planned dialysis on HRQOL or depression in patients with ESRD has not yet been investigated thoroughly. Planned dialysis was found to be beneficial in 2 previous studies. However, the study sample sizes were small, and one of them only included old patients [10,11]. Only 3 studies have reported data on the relationship between referral timing and HRQOL, but their results are conflicting [10,12,13]. Moreover, Beck’s Depression Inventory (BDI) according to the referral timing or planned start was rarely studied and it was shown in only one small study [14].

Therefore, we investigated the effect of referral timing and planned dialysis on HRQOL and depression in patients with ESRD in a nationwide prospective cohort in Korea. We aimed to examine whether nephrologists play a positive role in improving the future HRQOL and depression in early referral CKD patients.

## Subjects and Methods

### Study Design and Definition

This study was planned as a component of a comprehensive prospective study of the Clinical Research Center for End Stage Renal Disease (CRC for ESRD) in Korea. It was a nationwide, prospective cohort study of patients with ESRD initiated on dialysis therapy.

Patients were classified as early referral if their first encounter with a nephrologist occurred more than 1 year before initiation of dialysis and received education about dialysis (education from a nurse or nephrologist), and all others were classified as late referral, as described previously [7].

Planned dialysis was defined as dialysis therapy initiated with a permanent access (i.e., a peritoneal dialysis [PD] catheter for PD or either an arteriovenous graft or a fistula for hemodialysis [HD]) and education about dialysis, and the rest was considered as unplanned dialysis.

### Study Population

Patients with ESRD who were aged ≥ 20 years and were initiated on dialysis therapy were enrolled in the CRC for ESRD. It was a nationwide web-based, multicenter, joint-network, prospective cohort study of patients with ESRD in Korea and was designed to improve the survival rates and quality of life (QOL) in patients with ESRD and to create effective treatment guidelines [7,15]. Thirty-one hospitals and clinics in Korea participated in the CRC for ESRD and shared the clinical data of 1,270 newly diagnosed adult patients with ESRD who are supposed to continue dialysis at least 3 months between August 2008 and June 2012. Of these, patients who completed questionnaires at 3 months after dialysis were enrolled in this study. All patients provided their written consent to participate in this study. All traceable identifiers were removed before analysis to protect patient confidentiality. This study was approved by the institutional review board at each center [The Catholic University of Korea, Bucheon St. Mary’s Hospital; The Catholic University of Korea, Incheon St. Mary’s Hospital; The Catholic University of Korea, Seoul St. Mary’s Hospital; The Catholic University of Korea, St. Mary’s Hospital; The Catholic University of Korea, St. Vincent’s Hospital; The Catholic University of Korea, Uijeongbu St. Mary’s Hospital; Cheju Halla General Hospital; Chonbuk National University Hospital; Chonnam National University Hospital; Chung-Ang University Medical Center; Chungbuk National University Hospital; Chungnam National University Hospital; Dong-A University Medical Center; Ehwa Womens University Medical Center; Fatima Hospital, Daegu; Gachon University Gil Medical Center; Inje University Pusan Paik Hospital; Kyungpook National University Hospital; Kwandong University College of Medicine, Myongji Hospital; National Health Insurance Corporation Ilsan Hospital; National Medical Center; Pusan National University Hospital; Samsung Medical Center, Seoul; Seoul National University, Boramae Medical Center; Seoul National University Hospital; Seoul National University, Bundang Hospital; Yeungnam University Medical Center; Yonsei University, Severance Hospital; Yonsei University, Gangnam Severance Hospital; Ulsan University Hospital; Wonju Christian Hospital (in alphabetical order)]. All clinical investigations were conducted in accordance with the guidelines of the 2008 Declaration of Helsinki.

### Measurements

As sociodemographic and socioeconomic characteristics, age, sex, marital status (single, married, divorced/separated/widowed), educational attainment, and occupation were considered in the analysis. Educational level was assessed as the number of years of schooling. For the analysis, three groups were defined: 9 years, 10–12 years, and ≥ 13 years of schooling. In Korea, 9 years, 10–12 years, and ≥ 13 years of schooling are equivalent to elementary/middle school, high school, and college/university or above, respectively. Occupation was also classified into three groups as categorical variables in this study: jobless, housewife, and employed. The estimated glomerular filtration rate (eGFR) was calculated by CKD-EPI equations [16].

Kidney Disease Quality of Life Short Form 36 (KDQOL-36) has been used to evaluate the HRQOL of patients with ESRD [17]. We utilized the Korean version of the KDQOL-36, which was recently translated and validated in Korean patients with ESRD [18]. Briefly, this includes 12 items that provide a generic chronic disease core as well as 24 additional kidney disease-targeted items. The 24 additional items focus on particular health-related concerns of individuals with kidney disease (i.e., symptom/problem list, 12 items; effects of kidney disease, 8 items; and burden of disease, 4 items). The item scores were aggregated without weighting and transformed linearly to a 0–100 possible range, with higher scores indicating better states, which resulted in a total of dimensions.

The Korean version of BDI was used to evaluate depression in our patients. The BDI has been validated in various groups of patients and has been used in patients undergoing dialysis to evaluate depression [19]. It consists of 21 self-reported items, and each item is rated on a scale of 0 to 3, producing a possible score range of 0 to 63 [20].

Patients who completed two surveys at 3 months after dialysis were included in this study, and some of these patients completed these surveys again at 1 year after dialysis.

### Statistical analysis

First, the overall distributions of the characteristics of the respondents were presented as frequencies and percentages for categorical variables, mean ± SD for continuous variables, or median values (25% and 75% interquartile ranges) for non-normally distributed variables, according to the type of plan.

We performed univariate analyses by using the *t*-test to compare the differences in the KDQOL-36 and BDI scores at after 3 months and 1 year after dialysis, according to the type of plan and referral, respectively. In addition to univariate analyses, multivariate regression analyses were applied for each component of KDQOL-36 and BDI scores including type of plan as an independent variable. In multiple regression models, we adjusted for age (continuous), sex, type of dialysis, marital status, educational attainment, occupation, modified Charlson comorbidity index (MCCI, continuous), albumin (continuous), and hemoglobin (continuous) levels. All continuous covariates were centered at the mean of each variable. All statistical analyses were performed using SAS version 9.3 (SAS Institute, Cary, NC, USA) and R statistical language (Version R 3.0.2, The Comprehensive R Archive Network: http://cran.r-project.org).

## Results

Among 1,270 patients, there were 1,026 patients whose age, sex, and dialysis modality were identified. Of 1,026 patients, 670 (65.3%) patients completed the questionnaire at 3 months after dialysis. Excluding 27 patients with no information on referral timing, planned dialysis a total of 643 patients were analyzed in the present study.

### Characteristics of analyzed patients compared to the entire cohort

As the questionnaire response rate was 65.3%, we compared baseline characteristics of our analyzed 643 patients with entire cohort 1,026 patients to evaluate selection bias (Table 1). There was no difference in age, sex and type of dialysis between the entire cohort and analyzed patients. Though referral timing, planned dialysis, social factors and cause of ESRD seemed to be different, it might be due to the missing data because the proportions were similar except missing data. Comorbidities, MCCI score and laboratory values were also similar between two groups. Therefore, we consider our analyzed 643 patients can represent the entire cohort.

**Table 1:** Baseline characteristics of entire cohort and analyzed patients according to planned vs. unplanned dialysis.

	Total (N = 1026)	Study population (N = 643)	p Value^†^	Planned (N = 348)	Unplanned (N = 295)	p Value^†^
	Mean ± SD or N (%)*	Mean ± SD or N (%)*		Mean ± SD or N (%)*	Mean ± SD or N (%)*	
Age (years)	55.9 ± 14.2	54.8 ± 13.5	0.130	54.0 ± 12.6	55.8 ± 14.5	0.086
Sex, Male	608 (59.3)	380 (59.1)	0.950	204 (58.6)	176 (59.7)	0.789
Type of dialysis						
Hemodialysis	687 (67.0)	421 (65.5)	0.530	145 (41.7)	276 (93.6)	<0.001
Peritoneal dialysis	339 (33.0)	222 (34.5)		203 (58.3)	19 (6.4)	
Type of referral						
Early referral	582 (56.7)	390 (60.7)	<0.001	232 (66.7)	158 (53.6)	0.001
Late referral	382 (37.2)	253 (37.4)		116 (33.3)	137 (46.4)	
Missing	62 (6.0)	0 (0)		0 (0)	0 (0)	
Number of visit						
None	60 (5.8)	31 (4.8)	<0.001	0 (0)	32 (14.2)	<0.001
1 time	71 (6.9)	55 (8.6)		27 (7.7)	195 (66.1)	
≥ 2 times	845 (82.4)	550 (85.5)		315 (90.5)	51 (17.3)	
Missing	50 (4.9)	7 (1.1)		6 (1.7)	7 (2.4)	
Marital status						
Single	117 (11.4)	81 (12.6)	<0.001	39 (11.2)	42 (14.2)	0.293
Married	706 (68.8)	447 (69.5)		252 (72.4)	195 (66.1)	
Divorced/separated/widowed	128 (12.5)	103 (16.0)		52 (14.9)	51 (17.3)	
Missing	75 (7.3)	12 (1.9)		5 (1.4)	7 (2.4)	
Educational attainment (years)						
≤ 9	367 (35.8)	254 (39.5)	<0.001	140 (40.2)	114 (38.6)	0.068
10–12	318 (31.0)	219 (34.1)		105 (30.2)	114 (38.6)	
> 13	270 (26.3)	165 (25.6)		99 (28.4)	66 (22.4)	
Missing	71 (6.9)	5 (0.8)		4 (1.1)	1 (0.3)	
Occupation						
Jobless including students	497 (48.5)	316 (49.1)	<0.001	166 (47.7)	150 (50.8)	0.549
Housewife	193 (18.8)	126 (19.6)		73 (21.0)	53 (18.0)	
Employed (waged)	273 (26.6)	191 (29.7)		102 (29.3)	89 (30.2)	
Missing	63 (6.1)	10 (1.6)		7 (2.0)	3 (1.0)	
Cause of ESRD						
Diabetes mellitus	590 (57.5)	378 (58.8)	0.002	196 (56.3)	182 (61.7)	0.405
Hypertension	156 (15.2)	90 (14.0)		46 (13.2)	44 (14.9)	
Glomerulonephritis	159 (15.5)	121 (18.8)		72 (20.7)	49 (16.6)	
Others	107 (10.4)	37 (5.8)		23 (6.6)	14 (4.7)	
Missing	14 (1.4)	17 (2.6)		11 (3.2)	6 (2.0)	
Score of MCCI	5.17 ± 2.34	5.03 ± 2.54	0.27	5.07 ± 2.61	4.99 ± 2.46	0.683
Comorbidities						
Diabetes mellitus	575 (56.0)	367 (57.1)	0.653	195 (56.0)	172 (58.8)	0.529
Coronary artery disease	130 (12.7)	85 (13.2)	0.709	51 (14.7)	34 (11.7)	0.271
Cerebrovascular disease	107 (10.4)	71 (11.0)	0.661	40 (11.5)	31 (10.6)	0.715
Congestive heart failure	129 (12.6)	97 (15.1)	0.138	56 (16.1)	41 (14.0)	0.460
Malignancy	75 (7.3)	43 (6.7)	0.653	24 (6.9)	19 (6.5)	0.820
Laboratory values						
Cr at referral (mg/dL)	5.18 ± 10.0	5.12 ± 10.0	0.910	4.24 ± 3.8	5.27 ± 4.6	0.006
eGFR at referral (ml/min/1.73 m^2^)	23.61 ± 21.5	24.22 ± 22.7	0.621	24.66 ± 22.4	23.72 ± 23.0	0.639
Cr at start of dialysis (mg/dL)	8.64 ± 6.1	8.79 ± 6.7	0.643	8.58 ± 7.9	9.04 ± 5.0	0.386
eGFR at start of dialysis (ml/min/1.73 m^2^)	7.34 ± 5.9	7.15 ± 5.5	0.521	7.43 ± 6.7	6.82 ± 3.6	0.162
Albumin (g/dL)	3.35 ± 0.61	3.35 ± 0.58	0.85	3.35 ± 0.58	3.34 ± 0.58	0.748
Hemoglobin (g/dL)	8.94 ± 1.67	8.81 ± 1.70	0.13	9.05 ± 1.56	8.51 ± 1.83	<0.001

SD, standard deviation; ESRD, end-stage renal disease; MCCI, modified Charlson comorbidity index; Cr, creatinine; eGFR, estimated glomerular filtration rate.

*Values are presented as n (%) for categorical variables, mean ± SD for continuous variables.

†p value was obtained from bivariate analysis.

### Baseline characteristics

Of the 643 patients, 348 (54.1%) and 295 (45.9%) patients were included in the planned and unplanned dialysis groups, respectively (Table 1). Early referral was significantly dominant in the planned group. The mean age was 54.8 years, and 59.1% of patients were male. The proportion of PD was much higher in planned group. Social variables such as marital status, educational attainment, and occupation did not differ between the two groups. Clinical variables such as comorbidities and laboratory values were similar, except hemoglobin levels, which were higher in the planned group.

Among 643 study population, there were 95 patients who dropped out in 1 year due to hospital transfer (45.3%), transplantation (15.8%), death (14.7%), and refusal (7.4%). Finally, 291 patients completed the follow-up surveys at 1 year after dialysis.

### KDQOL-36 and BDI: effect of referral type and planned dialysis

On comparing the KDQOL-36 and BDI scores on the basis of the referral type, no difference was observed between the groups both at 3 months and 1 year after dialysis (Table 2). However, the planned dialysis group showed significantly higher scores in 4 of 5 KDQOL-36 domains than did the unplanned group at 3 months after dialysis (Table 3). Even after adjusting for confounding variables including age, sex, type of dialysis, marital status, educational attainment, occupation, MCCI, hemoglobin, and albumin level, the planned group showed higher scores in 3 domains (mental component score, symptom/problem list, and effect of disease). After adjusting for confounding variables, BDI scores at 3 months after dialysis were also significantly lower in planned dialysis group which indicate less depressive mood.

**Table 2:** Group comparisons of KDQOL-36 and BDI scores (early *vs*. late referral).

	Total Mean (SD)	Early referral Mean (SD)	Late referral Mean (SD)	p Value^*^
At 3 months after dialysis				
KDQOL-36				
PCS	40.4 (9.4)	40.3 (9.3)	40.4 (9.6)	0.810
MCS	40.5 (10.0)	40.8 (10.1)	40.0 (9.9)	0.349
Symptom/problem list	80.0 (15.8)	80.3 (15.9)	79.4 (15.6)	0.491
Effect of disease	69.6 (18.8)	69.7 (18.5)	69.5 (19.1)	0.892
Burden of disease	33.7 (22.6)	33.8 (22.6)	33.6 (22.6)	0.916
15.4 (10.6)	15.5 (11.0)	15.2 (10.0)	0.714
At 1 year after dialysis				
KDQOL-36				
PCS	42.3 (9.3)	42.2 (9.1)	42.5 (9.7)	0.799
MCS	41.5 (9.0)	41.9 (8.8)	40.8 (9.4)	0.316
Symptom/problem list	82.3 (13.9)	82.0 (13.6)	83.0 (14.5)	0.548
Effect of disease	72.6 (17.9)	72.0 (17.3)	73.7 (18.9)	0.438
Burden of disease	35.3 (24.0)	36.1 (23.8)	33.8 (24.4)	0.424
BDI	14.6 (10.0)	14.2 (9.8)	15.3 (10.5)	0.358

SD, standard deviation; KDQOL-36, Kidney Disease Quality of Life Short Form 36; PCS, physical component summary; MCS, mental component summary; BDI, Beck’s Depression Inventory.

*p value was obtained using the *t*-test.

**Table 3:** Group comparisons of KDQOL-36 and BDI scores (planned *vs*. unplanned dialysis patients).

	Total Mean (SD)	Planned Mean (SD)	Unplanned Mean (SD)	p Value^*^	p Value^†^
At 3 months after dialysis					
KDQOL-36					
PCS	40.4 (9.4)	41.2 (9.5)	39.5 (9.3)	0.027	0.103
MCS	40.5 (10.0)	41.2 (9.9)	39.7 (10.1)	0.060	0.014
Symptom/problem list	80.0 (15.8)	81.6 (15.0)	78.1 (16.4)	0.006	0.006
Effect of disease	69.6 (18.8)	71.5 (17.3)	67.4 (20.1)	0.005	0.032
Burden of disease	33.7 (22.6)	35.8 (22.6)	31.3 (22.3)	0.012	0.273
BDI	15.4 (10.6)	15.0 (10.1)	15.8 (11.2)	0.317	0.047
At 1 year after dialysis					
KDQOL-36					
PCS	42.3 (9.3)	43.2 (9.3)	41.0 (9.2)	0.058	0.004
MCS	41.5 (9.0)	42.1 (9.0)	40.5 (8.9)	0.142	0.157
Symptom/problem list	82.3 (13.9)	84.2 (12.8)	79.8 (15.2)	0.016	0.008
Effect of disease	72.6 (17.9)	74.2 (16.8)	70.11 (19.3)	0.065	0.384
Burden of disease	35.3 (24.0)	37.1 (23.6)	32.5 (24.4)	0.115	0.535
BDI	14.6 (10.1)	13.4 (9.4)	16.3 (10.8)	0.015	0.038

SD, standard deviation; KDQOL-36, Kidney Disease Quality of Life Short Form 36; PCS, physical component summary; MCS, mental component summary; BDI, Beck’s Depression Inventory.

*p value was obtained using the *t*-test.

†p value was obtained from regression analysis adjusted for age, sex, type of dialysis, marital status, educational attainment, occupation, modified Charlson comorbidity index, albumin, and hemoglobin levels. Type of plan was included as independent variable.

At 1 year, all the KDQOL-36 and BDI scores slightly increased compared to those at 3 months after dialysis (Table 3). The planned group had higher mean scores in each KDQOL-36 domain than did the unplanned group, and this difference was statistically significant in 1 domains. After adjusting for confounding variables, physical component score and symptom/problem list domain were significant higher in planned dialysis group. Mean BDI scores were 13.4 and 16.3 in planned and unplanned groups, respectively. The difference was significant before and after adjustment that indicates planned dialysis patients are less depressive at 1year after dialysis.

Next, to exclude the effect of dialysis modality, we conducted subgroup analysis only including HD patients. As shown in Table 4, several domains of KDQOL-36 and BDI were still significantly better in planned dialysis group both at 3 months and 1 year after dialysis.

Finally, one year follow-up questionnaire was only completed by 291 (45.3%) among 643 patients. We compared KDQOL-36 and BDI at 3 months after dialysis between the follow-up group and no follow-up group, which showed no difference between the groups (S1 Table).

**Table 4:** Group comparisons of KDQOL-36 and BDI scores only in hemodialysis patients (planned *vs*. unplanned dialysis patients).

	Total Mean (SD)	Planned Mean (SD)	Unplanned Mean (SD)	p Value^*^	p Value^†^
At 3 months after dialysis					
KDQOL-36					
PCS	39.7 (9.4)	40.7 (9.4)	39.2 (9.3)	0.121	0.042
MCS	40.6 (9.8)	42.3 (9.0)	39.7 (10.1)	0.012	0.006
Symptom/problem list	79.2 (16.0)	82.2 (14.6)	77.7 (16.6)	0.004	0.001
Effect of disease	68.3 (18.8)	70.0 (15.9)	67.4 (20.2)	0.148	0.087
Burden of disease	31.5 (21.5)	32.8 (20.0)	30.8 (22.2)	0.370	0.102
BDI	15.2 (10.8)	13.7 (9.4)	16.0 (11.3)	0.031	0.012
At 1 year after dialysis					
KDQOL-36					
PCS	42.3 (9.5)	45.1 (9.3)	40.2 (9.1)	0.001	0.001
MCS	41.5 (8.8)	43.0 (8.2)	40.5 (9.1)	0.062	0.180
Symptom/problem list	82.3 (14.1)	85.9 (11.9)	79.7 (15.1)	0.003	0.022
Effect of disease	70.5 (18.1)	71.8 (16.6)	69.6 (19.1)	0.440	0.597
Burden of disease	32.9 (23.7)	34.3 (22.3)	31.8 (24.7)	0.485	0.487
BDI	15.0 (10.2)	12.5 (9.2)	16.8 (10.6)	0.005	0.031

SD, standard deviation; KDQOL-36, Kidney Disease Quality of Life Short Form 36; PCS, physical component summary; MCS, mental component summary; BDI, Beck’s Depression Inventory.

*p value was obtained using the *t*-test.

†p value was obtained from regression analysis adjusted for age, sex, marital status, educational attainment, occupation, modified Charlson comorbidity index, albumin, and hemoglobin levels. Type of plan was included as independent variable.

## Discussion

We evaluated the HRQOL and depression of patients undergoing dialysis according to the referral timing and planned initiation. In this study, not early referral but planned dialysis was found to improve the HRQOL and depression.

Planned dialysis can reduce hospitalization duration, economic costs, and mortality in patients with ESRD [21]. Only two studies have reported that QOL was better when the patient started dialysis in a planned manner. Caskey et al. studied the effect of planned dialysis on QOL in patients with ESRD between 1998 and 1999 in seven European countries by using the visual analogue scale and Short Form-36 [10]. The planned dialysis group showed higher visual analogue scale scores, MCS score, role emotional scores, and mental health scores in Short Form-36. However, this analysis included only 196 patients who were referred early, and planned dialysis initiation was determined on the basis of the last serum creatinine level and emergent clinical events. Another small cross-sectional study evaluated only patients aged ≥ 70 years [11]. In this study, the planned dialysis group also had a better QOL than did the unplanned group and had a similar QOL as that in the non-ESRD age- and sex-matched control group. Our study showed results consistent with those reported by these two previous studies.

Furthermore, we conducted subgroup analysis including only hemodialysis patients to exclude the effect of dialysis modality which was significantly dominant in planned group. The results clearly showed the benefit of planned dialysis PD on patients’ QOL without doubt.

BDI have been used to evaluate depression in ESRD patients in many previous studies. [1,2]. However, BDI according to referral timing or planned dialysis was rarely investigated. In one small study showed that among incident dialysis patients, depressive patients are consisted with more proportion of unplanned starters who had no contact with a nephrologist within 90 days [14]. To the best of our knowledge, this is the only report that comprehensively elucidated the difference of BDI according to planned dialysis.

Our results are significant because the benefit of planned dialysis on HRQOL and depression was proven in a large-scale, multicenter, prospective ESRD cohort study. We used the KDQOL-36 survey, which contains kidney disease-specific questions. Moreover, we first observed that this beneficial effect sustained until 1 year after dialysis while previous studies only evaluated QOL at the initiation or 8 weeks of dialysis.

Regarding patient characteristics, the planned dialysis group showed higher hemoglobin levels. Although age and albumin levels did not differ significantly between the two groups, age was younger and albumin was higher in the planned group. These findings are consistent with those of the previous reports [22,23]. Regarding the dialysis modality, planned dialysis is associated with more use of PD as it was shown in the previous study [23]. Socioeconomic factors did not differ between the planned and unplanned groups. In fact, referral type was almost the only modifiable factor for planned dialysis.

As shown in previous and the present studies, early referral patients have a better chance of initiating dialysis in a planned manner with permanent vascular access [6,7]. The benefit of planned dialysis might be attributable to predialytic education, psychosocial preparation for dialysis, and no catheterization. Likewise, early referral itself is expected to have similar benefits because it allows the patient more time to discuss renal progression and dialysis modalities with their nephrologists. However, surprisingly, referral type did not affect QOL in dialysis patients. In other words, early referral patients did not benefit if they initiated dialysis in an unplanned manner. The STARRT (Study To Assess Renal Replacement Therapy) study reported similar results related to the composite outcome of death, transfusion and hospitalization instead of QOL [9]. This fact raises two points of criticism against nephrologists.

First, unplanned dialysis is performed quite commonly in early referral patients. In the present study, the incidence of unplanned dialysis was 40.8% in early referral patients. In previous STARRT study, the rate of unplanned dialysis in early referral was very similar with ours [9]. In the study, optimal start defined if all the following were true; (i) RRT initiated as an outpatient and (ii) dialysis was initiated with a permanent access. Among early referral patients, suboptimal starts occurred in 44% which was higher than expected. Hughes et al. reported that factors contributing to suboptimal starts despite early referral were patient-related delays (31.2%), acute-on-chronic kidney disease (31.2%), surgical delays (16.4%), and late decision-making (8.59) [24]. The patient-related delays and late decision-making seems modifiable by nephrologists. Patients are usually reluctant to undergo dialysis, but nephrologists should be aware of the benefits of the planned dialysis and assume the responsibility to educate and persuade them to undergo planned dialysis. Moreover, nephrologists should be careful to avoid making late decisions regarding dialysis access formation and dialysis initiation.

Second, nephrologists should make an effort to improve patient QOL, apart from providing planned dialysis. Providing multidisciplinary care is a good example. Kidney Disease: Improving Global Outcomes 2012 clinical practice guidelines for evaluating and managing CKD also suggest that patients with progressive CKD should be managed in a multidisciplinary care setting that includes dietary counseling, education of dialysis modalities and transplant options, vascular access surgery, and psychosocial care [25].

Our study has a limitation. Only 291 of 643 patients completed the follow-up surveys at 1 year after dialysis. We believe that if more patients were analyzed, the positive effect of planned dialysis would be much clearer.

Taken together, planned initiation of dialysis significantly improved HRQOL and depression in patients with ESRD. However, early referral did not show the same benefits. We suggest that nephrologists should try to persuade and educate patients to start dialysis in a planned manner and provide multidisciplinary care to improve their QOL.

## Supporting Information

S1 Table Group comparisons of KDQOL-36 and BDI scores (follow-up group *vs*. no follow-up group). (DOCX)
