# Supplementary material for: Not Early Referral but Planned Dialysis Improves Quality of Life and Depression in Newly Diagnosed End Stage Renal Disease Patients: A Prospective Cohort Study in Korea
Source: PLoS One. 2015 Feb 23;10(2):e0117582. doi: 10.1371/journal.pone.0117582 (PMC4338188; doi:10.1371/journal.pone.0117582)
Supplement: S1 Table — (DOCX) [file pone.0117582.s001.docx]

**S1 Table. Group comparisons of KDQOL-36 and BDI scores (follow-up group *vs.* no follow-up group)**

|  |  | Total Mean (SD) | | Follow-up  N=291 Mean (SD) | | No follow-up  N=352  Mean (SD) | | p Value^*^ | p Value^†^ |
| --- | --- | --- | --- | --- | --- | --- | --- | --- | --- |
| KDQOL-36 | |  | |  | |  | |  |  |
|  | PCS | 40.4 (9.4) | | 40.0 (9.8) | | 40.4 (9.1) | | 0.295 | 0.153 |
|  | MCS | 40.5 (10.0) | | 39.9 (10.4) | | 41.0 (9.7) | | 0.169 | 0.383 |
|  | Symptom/problem list | 80.0 (15.8) | | 80.5 (14.5) | | 79.6 (16.8) | | 0.468 | 0.897 |
|  | Effect of disease | 69.6 (18.8) | | 68.6 (19.2) | | 70.5 (18.4) | | 0.213 | 0.173 |
|  | Burden of disease | 33.7 (22.6) | | 32.2 (23.6) | | 35.0 (21.6) | | 0.119 | 0.141 |
| BDI | | 15.4 (10.6) | | 15.8 (9.7) | | 15.0 (11.3) | | 0.315 | 0.497 |
|  | |  |  | |  | |  |  |  |

SD, standard deviation; KDQOL-36, Kidney Disease Quality of Life Short Form 36; PCS, physical component summary; MCS, mental component summary; BDI, Beck’s Depression Inventory.

*p value was obtained using the *t*-test.

†p value was obtained from regression analysis adjusted for age, sex, type of dialysis, marital status, educational attainment, occupation, modified Charlson comorbidity index, albumin, and hemoglobin levels. Follow-up was included as independent variable.
